# Recent Developments in Nanoparticle-Based siRNA Delivery for Cancer Therapy

**DOI:** 10.1155/2013/782041

**Published:** 2013-06-17

**Authors:** Jong-Min Lee, Tae-Jong Yoon, Young-Seok Cho

**Affiliations:** ^1^Department of Internal Medicine, Uijeongbu St. Mary's Hospital, The Catholic University of Korea College of Medicine, Uijeongbu 480717, Republic of Korea; ^2^Department of Applied BioScience, CHA University, Sungnam 463836, Republic of Korea

## Abstract

RNA interference (RNAi) is a gene regulation mechanism initiated by RNA molecules that enables sequence-specific gene silencing by promoting degradation of specific mRNAs. Molecular therapy using small interfering RNA (siRNA) has shown great therapeutic potential for diseases caused by abnormal gene overexpression or mutation. The major challenges to application of siRNA therapeutics include the stability and effective delivery of siRNA *in vivo*. Important progress in nanotechnology has led to the development of efficient siRNA delivery systems. In this review, the authors discuss recent advances in nanoparticle-mediated siRNA delivery and the application of siRNA in clinical trials for cancer therapy. This review will also offer perspectives on future applications of siRNA therapeutics.

## 1. Introduction

 RNA interference (RNAi) is a process by which RNA molecules, with sequences complementary to a gene's coding sequence, induce degradation of corresponding messenger RNAs (mRNAs), thus blocking the translation of the mRNA into protein [1, 2]. RNAi is initiated by exposing cells to long dsRNA via transfection or endogenous expression. dsRNAs are processed into smaller fragments (usually 21–23 nucleotides) of small interfering RNAs (siRNA) [3], which form a complex with the RNA-induced silencing complexes [4]. Introduction of siRNA into mammalian cells leads to downregulation of target genes without triggering interferon responses [3]. Molecular therapy using siRNA has shown great potential for diseases caused by abnormal gene overexpression or mutation, such as various cancers, viral infections, and genetic disorders, as well as for pain management. In the last 10 years, a tremendous effort has been made in biomedical therapeutic application of gene silencing in humans. Phase I studies of siRNA for the treatment of age-related macular degeneration and respiratory syncytial virus provided promising data with no sign of nonspecific toxicity [5, 6]. However, there are many challenges to be overcome for siRNA cancer therapeutics, including safety, stability, and effective siRNA delivery.

 The major barrier facing siRNA therapeutics is the efficiency of delivery to the desired cell type, tissue, or organ. siRNAs do not readily pass through the cell membrane due to their size and negative charge. Cationic liposome-based strategies are usually used for the cellular delivery of chemically synthesized or *in vitro* transcribed siRNA [7]. However, there are many problems with lipid-based delivery systems *in vivo*, such as rapid clearance by the liver and lack of target tissue specificity. Delivery systems can be categorized into physical methods, conjugation methods, and natural carrier (viruses and bacteria) and nonviral carrier methods [8]. DNA-based expression cassettes that express short hairpin RNA (shRNA) are usually delivered to target cells *ex vivo* by viruses and bacteria, and these modified cells are then reinfused back into the patient [9]. The popular adenovirus- and adeno-associated virus-derived vectors provide efficient delivery for shRNA expression [10]. However, there are problems with delivery using viral vectors, such as insertional mutagenesis and immunogenicity [11]. Nonviral gene delivery systems are highly attractive for gene therapy because they are safer and easier to produce than viral vectors. Nanotechnology has made significant advances in the development of efficient siRNA delivery systems. Current nonviral delivery systems can be categorized as organic and inorganic [12]. Organic complexes include lipid complexes, conjugated polymers, and cationic polymers, whereas inorganic nanoparticles include magnetic nanoparticles, quantum dots, carbon nanotubes, and gold nanoparticles. In this review, the authors discuss recent advances in nanoparticle-mediated siRNA delivery systems and the application of these systems in clinical trials for cancer therapy. Furthermore, we offer perspectives on future applications of siRNA therapeutics.

## 2. Lipid-Based Nanovectors for Systemic siRNA Delivery

### 2.1. Liposomes/Lipoplexes

Liposomes/lipoplexes have been extensively explored as nonviral vectors for plasmid and siRNA delivery [13]. Lipoplexes are complexes between cationic lipids and nucleic acids (mainly as plasmid DNA) [14]. Although neutral liposomes are more biocompatible than cationic lipids and have superior pharmacokinetics, they have low entrapment efficiency due to the lack of interaction between neutral lipids and anionic polynucleotides during formulation [15]. To increase entrapment efficiency, Landen Jr. et al. developed a method of formulating 1,2-dioleoyl-sn-glycero-3-phosphatidylcholine- (DOPC-) encapsulated siRNA liposomes that involves dissolving DOPC and siRNA in excess tertiary-butanol in the presence of the nonionic detergent Tween 20 [16]. DOPC-encapsulated siRNA targeting the oncoprotein EphA2 was highly effective in reducing EphA2 expression 48 h after administration of a single dose in an orthotopic model of ovarian carcinoma [16]. Treatment with DOPC-encapsulated siRNA via intravenous or intraperitoneal injections was highly effective in reducing both *in vivo* expression of target genes (e.g., EphA2, FAK, neuropilin-2, or IL-8) and tumor weight in mouse models of different human cancers [16–19]. In 2012, M.D. Anderson Cancer Center initiated a phase I dose-escalation trial for neutral liposome (DOPC) targeting of Eph2 in patients with advanced, recurrent cancer (http://www.clinicaltrials.gov/ct2/show/NCT01591356).

Cationic lipids, such as dioleoyl phosphatidylethanolamine and 1,2-dioleoyl-3-trimethylammonium-propane (DOTAP), form lipoplexes with negatively charged siRNA [15, 20]. Cationic liposomes are routinely used for delivery of siRNA or plasmid DNA into mammalian cells *in vitro* [21]. However, surface interactions of cationic liposomes with the tumor cells produce an electrostatically derived binding site barrier effect, inhibiting further association of the delivery systems with tumor spheroids [22]. In addition, although cationic liposomes efficiently take up siRNA, limited success has been achieved with these systems in *in vivo* gene silencing, probably due to their intracellular stability and resultant failure to release siRNA contents [20]. Finally, the effectiveness of cationic liposomes has been limited by their toxicity. The use of cationic liposomes *in vivo* elicited dose-dependent toxicity and pulmonary inflammation by promoting release of reactive oxygen intermediates [23–25]. This effect was more pronounced with the multivalent cationic liposomes than with the monovalent cationic lipids, such as DOTAP [24].

The coating of liposomes with hydrophilic molecules, such as polyethylene glycol (PEG), reduced uptake by the reticuloendothelial system (RES), resulting in enhanced circulatory half-life [26]. In 2006, Santel et al. developed a novel liposomal siRNA formulation based on cationic lipids (siRNA-lipoplex/AtuPLEX), containing neutral fusogenic and PEG-modified lipid components, for improved pharmacokinetics and cellular uptake, and more efficient siRNA release [27, 28]. Using this formulation to target endothelia-specific genes, such as CD31 (platelet endothelial cell adhesion molecule-1) or TIE-2, they demonstrated downregulation of the corresponding mRNAs and proteins in mice [28]. Atu027 is a lipoplexed siRNA molecule specifically targeting the expression of protein kinase N3, which has been identified as a downstream effector of the phosphoinositol-3-kinase signaling pathway [29]. Atu027 has been reported to inhibit lymph node metastasis in orthotopic prostate and pancreatic cancer mouse models and to inhibit hematogenous metastasis to the target organ lung in various mouse lung metastasis models [29, 30]. Silence Therapeutics (London, UK) is performing a phase I trial of Atu027, which was well tolerated up to a dose of 0.180 mg/kg and was not associated with dose-dependent toxicities, in patients with colorectal cancer metastasized to the liver [31]. Dose escalation is currently being investigated. Using liposomal encapsulation of siRNA nanoparticles, another delivery platform, tauRNAi, has been developed by Marina Biotech (Bothell, WA, USA). This drug is in the preclinical stage for hepatocellular carcinoma [32].

### 2.2. Stable Nucleic Acid Lipid Particles and Lipidoids

Solid lipid-based systems have been developed as alternatives to emulsions, liposomes, microparticles, and polymeric nanoparticles for systemic delivery of siRNA and include stable nucleic acid lipid particles (SNALPs) and cationic solid-lipid nanoparticles [33, 34]. Jeffs et al. developed a new “spontaneous vesicle formation” method for the preparation of rapid and reproducible stabilized plasmid lipid particles for nonviral, systemic gene therapy [35]. Using this controlled, stepwise dilution method, Morrissey et al. developed SNALPs, which are PEG-conjugated lipid nanoparticles comprised of siRNA encapsulated inside a lipid bilayer of neutral lipids and PEG-lipid fusion regulators [33]. Stabilized siRNA targeting hepatitis B virus (HBV) RNA was incorporated into SNALPs and administered by intravenous injection into mice carrying replicating HBV, resulting in reduction of the level of HBV DNA. Furthermore, reductions were seen in serum HBV DNA for up to 6 weeks with weekly dosing. Zimmermann et al. have demonstrated that intravenous injection of ApoB-targeting siRNAs encapsulated in SNALPs resulted in significant dose-dependent silencing *ApoB* mRNA in the livers of both mice and nonhuman primates [36]. A single administration of 2.5 mg/kg SNALP-formulated siRNA was well tolerated and reduced *ApoB* mRNA expression in the liver by up to 90%, lasting for 11 days at the highest siRNA dose. SNALP-formulated siRNA targeting the essential cell-cycle proteins polo-like kinase 1 (PLK1) and kinesin spindle protein (KSP) showed potent antitumor efficacy in both hepatic and subcutaneous tumor models [37]. Tekmira Pharmaceuticals Corporation (Burnaby, BC, Canada) initiated a phase I trial of SNALP-encapsulated siRNA targeting PLK1 (TKM 080301) in December 2010 (http://www.clinicaltrials.gov/ct2/show/NCT01262235). This is dose-escalation trial conducted at multiple clinical centers, designed to determine TKM 080301 safety, tolerability, and pharmacokinetics in adult patients with solid tumors or lymphomas that are refractory to standard therapy or for whom there is no standard therapy. Alnylam Pharmaceuticals (Cambridge, MA, USA) has developed SNALP-formulated siRNAs targeting vascular endothelial growth factor (VEGF) and KSP in ALN-VSP02, the first dual-targeted siRNA drug. In April 2009, a phase I dose-escalation trial was initiated (http://www.clinicaltrials.gov/ct2/show/NCT00882180). Interim data from pharmacodynamic measurements provided preliminary evidence of clinical activity for the treatment of advanced solid tumors with liver involvement. Additional results from the initial 28 patients in the first six-dose cohorts demonstrated that ALN-VSP02 was generally well tolerated at the highest dose (1.25 mg/kg) [38]. The study has not yet reached a maximum tolerated dose and the trial continues to enroll patients in a dose-escalating manner. In another phase I trial, several patients with stable disease have advanced to a multicenter, open label, extension study to collect long-term safety data (http://www.clinicaltrials.gov/ct2/show/NCT01158079).

 Lipidoid nanoparticles are lipid-like delivery molecules comprised of cholesterol and PEG-modified lipids specific for delivery of specific siRNA [38]. To improve SNALP-mediated delivery, Akinc et al. developed a new chemical method to allow rapid synthesis of a large library of lipidoids and tested their efficacy in siRNA delivery [39]. The leading candidate, the 98N_12_-5 lipidoid-based siRNA formulation, showed 75%–90% reduction in ApoB or FVII factor expression in hepatocytes in nonhuman primates and mice [39, 40]. This formulation facilitated gene silencing at orders-of-magnitude lower doses of siRNA than those required by the original SNALP formulation, resulting in reduced toxicity [41].

## 3. Polymeric Nanoparticles

Polymeric nanoparticles are solid, biodegradable, colloidal systems that have been widely investigated as drug or gene carriers [42]. Polymeric nanoparticles are classified into two major categories, natural polymers and synthetic polymers. Natural polymers for siRNA delivery include cyclodextrin, chitosan, and atelocollagen [12]. Of the synthetic polymers, polyethyleneimine (PEI), poly(dl-lactide-*co*-glycolide) (PLGA), and dendrimers have been intensively investigated [8].

### 3.1. Cyclodextrin Nanoparticle

Cyclodextrins are natural polymers generated during the bacterial digestion of cellulose and can form water-soluble inclusion complexes with small molecules and portions of large compounds [43]. Hu-Lieskoven et al. developed the cyclodextrin-containing polycation system for the targeted delivery of siRNA [44]. This system consists of a cyclodextrin-containing polymer, PEG for stability, and human transferrin as the targeting ligand for binding to transferrin receptors, which are often overexpressed on cancer cells. This targeted nanoparticle system, called CALLA-01, was developed for the first siRNA phase I trial by Calando Pharmaceuticals (Pasadena, CA, USA) [45]. The siRNA in CALLA-01 is designed to inhibit tumor growth via a mechanism to reduce expression of the M2 subunit of ribonucleotide reductase (R2). Patients with solid cancers refractory to standard-of-care therapies were administered targeted nanoparticles via IV infusion on days 1, 3, 8, and 10 of a 21-day cycle [45]. Successful delivery of targeted nanoparticles was confirmed by the presence of intracellular nanoparticles in tumor biopsies from melanoma patients after treatment. Furthermore, knockdown of the M2 subunit of R2 was confirmed in tumor biopsies from these patients by quantitative reverse transcription-polymerase chain reaction (qRT-PCR) and by immunohistochemical staining in the patients treated with the highest dosage. This study demonstrated that siRNA administered systemically to humans may result in specific gene inhibition by an RNAi-mediated mechanism of action.

### 3.2. Chitosan Nanoparticles

Chitosan, a type of naturally occurring polysaccharide, has been extensively studied for the delivery of plasmid DNA and siRNA *in vitro* and *in vivo* [46–48]. The advantages of chitosan include mucoadhesivity, biocompatibility, biodegradability, and low cost of production. However, results of studies of siRNA delivery have been inconsistent due to discrepancies between experiments [46, 49]. In addition, high molecular weight chitosans are cytotoxic, thus limiting their use in clinical trials [50]. They still also lack the buffering capacity needed for endosomolysis, which is essential to siRNA release from the endosome [51].

### 3.3. Polyethyleneimine

PEI, a commonly used cationic polymeric drug carrier with high transfection efficiency, has been widely investigated for siRNA delivery [8, 12, 51]. PEI forms small and compact structures, spontaneously forming polyplexes, with negatively charged siRNA through a simple and short polycation process [12]. The PEI-siRNA complexes protect siRNA from nuclease degradation, resulting in prolonged half-life. In addition, complete encapsulation of siRNA prevents avoid off-target effects such as immune activation by a toll-like receptor dependent mechanism [52]. However, PEI complexes have been associated with significant toxicity issues limiting their broad use in clinical trials [50]. Molecular mechanisms of PEI cytotoxicity include membrane damage and activation of a mitochondria-mediated apoptotic program due to PEI-induced channel formation in the outer mitochondrial membrane [53, 54].

### 3.4. PLGA

PLGA is a copolymer of glycolic acid and lactic acid and a US Food and Drug Administration-approved biodegradable polymer [55]. PLGA has been used as a nanocarrier for plasmid DNA and siRNA delivery in recent years. The advantages of PLGA-based siRNA delivery include high stability, facile cellular uptake by endocytosis, ability to target specific tissues or organs by adsorption or ligand binding, biodegradability, low toxicity, and sustained release characteristics [56]. However, PLGA could not be applied efficiently in siRNA delivery due to the lower electrostatic interaction between PLGA and siRNA and less efficient endosomal escape and release of siRNA [8, 56]. To overcome these problems, the surface of PLGA can be decorated with various cationic nanoparticles such as DOTAP, PEI, or polyamine [51].

### 3.5. Dendrimers

Dendrimers are synthetic, highly branched monodisperse, and usually highly symmetric, spherical macromolecules with three-dimensional nanometric structure. The unique structural features such as tunable structure and molecular size, large number of accessible terminal functional groups, and ability to encapsulate cargos add to their potential as drug carriers [57]. Polycationic dendrimers such as poly(amidoamine) (PAMAM) and poly(propylenimine) (PPI) dendrimers have been studied for siRNA delivery in recent years. PAMAM dendrimers have become the most used dendrimer-based carriers for gene delivery because of the ease of synthesis and commercial availability. However, PAMAMs were demonstrated to be cytotoxic, predominately related to apoptosis mediated by mitochondrial dysfunction [58]. Cytotoxicity can be reduced by various modifications without compromising gene silencing. Surface-modified and cationic PAMAM dendrimers show very low cytotoxicity, even at high concentrations and efficiently penetrated cancer cells *in vitro* [59]. PPI dendrimers were also used to formulate siRNA nanoparticles, and these nanoparticles showed efficient gene silencing [60]. Dendrimer-conjugated magnetofluorescent nanoworms (dendriworms) were developed to achieve siRNA delivery in a transgenic murine model of glioblastoma [61]. These siRNA-carrying dendriworms maximized endosomal escape to robustly produce protein target knockdown and were tolerated well in mouse brain.

## 4. Inorganic Nanoparticles

A number of inorganic nanoparticles have been emerging as potential siRNA delivery systems devised for simultaneous imaging and therapeutic purposes. They include carbon nanotubes (CNTs) and metals such as iron oxide, quantum dots (QDs), and gold. CNTs are nanomaterials, with interesting physical and chemical properties, and have recently emerged as a new option for cancer treatment, bioengineering, and gene therapy. It has been proposed that CNTs easily cross the plasma membrane and translocate directly into cytoplasm of target cells due to their nanoneedle structure, using an endocytosis-independent mechanism without inducing cell death [62, 63]. CNTs are classified as single-walled CNTs and multiwalled CNTs [64]. Several functionalized CNTs have been designed and tested for the purpose of siRNA delivery. Zhang et al. used single-walled CNTs functionalized with –CONH–(CH_2_)_6_–NH_3_^+^Cl^−^ as siRNA carriers [65]. They released the siRNA from the nanotube side-wall to silence telomerase reverse transcriptase expression, which considerably suppressed tumor growth. CNTs functionalized with amine-terminated PEG (phospholipid (PL)-PEG2000-NH_2_) were shown to be efficient in siRNA delivery into human T cells [66]. Ammonium-functionalized CNTs and dendron-CNTs have also been reported to be efficient in siRNA delivery with low cytotoxicity [67, 68]. A comparative study of antitumor activity of the proprietary cytotoxic siRNA sequence (siTOX) delivered either by cationic liposomes (DOTAP : cholesterol) or amino-functionalized MWNT-NH^+3^ in a human lung xenograft model demonstrated that only MWNT-NH^+3^ : siRNA complexes administered intratumorally could elicit delayed tumor growth and increased survival of xenograft-bearing animals [69]. However, several studies have discussed the potential toxicity of CNTs although the underlying mechanisms are uncertain [70, 71].

Magnetic nanoparticles, including superparamagnetic iron oxide nanoparticles (SPIOs) and magnetic iron tetroxide particles, emerged as feasible nanotheranostics for tumor imaging and drug delivery due to their distinct characteristics [72]. The large surface area of SPIOs makes their functional modification feasible, enabling the conjugation of targeting molecules, drugs, and imaging agents [73]. Moore and her colleagues reported the synthesis and characterization of a new dual-purpose probe for the simultaneous noninvasive imaging and delivery of siRNAs to tumors [74]. This probe consists of magnetic nanoparticles (SPIOs for magnetic resonance imaging) conjugated with Cy5.5 dye (for near-infrared fluorescence imaging (NIRF)) and myristoylated polyarginine peptide for membrane translocation. A nanoparticle probe (MN-NIRF-siSurvivin) targeting the antiapoptotic gene Birc5, which encodes survivin, significantly increased cancer cell apoptosis and necrosis *in vitro* and in xenograft mouse models. Therefore, use of MN-NIRF-siSurvivin conjugates combining siRNA delivery with a dual-imaging modality (magnetic resonance imaging and NIRF) was feasible for multimodality imaging and targeted gene delivery. Lee et al. developed manganese-doped magnetism-engineered iron oxide (MnMEIO) nanoparticles conjugated to a cancer-specific targeting moiety the Arg-Gly-Asp (RGD) peptide, which specifically binds to tumors expressing *α*_v_ *β*_3_ integrin, and Cy5-dye-labeled siGFP, which inhibits the expression of green fluorescence protein (GFP) [75]. The constructed nanoparticle (MnMEIO-siGFP-Cy5/PEG-RGD) showed specific internalization and target gene silencing in *α*_v_ *β*_3_ integrin-expressing breast cancer MDA-MB-435 cells. An additional advantage of iron oxide nanoparticle delivery systems is that they can be delivered in a targeted manner to a desired region by applying an external magnetic field [76]. 

 Semiconductor QDs, which are light-emitting nanoparticles, have been increasingly used as biological imaging and labeling probes [77]. QDs also have the potential of serving as photostable beacons for siRNA delivery and imaging [78–80]. However, the major problem in using QDs as multifunctional imaging probes and delivery systems is their toxicity because most well-established QDs are composed of highly toxic elements, such as cadmium, selenium, or tellurium [81]. Recently, nontoxic QDs, which were developed by a novel sonochemical approach for the high-throughput synthesis of a library of biocompatible ZnS-AgInS2 QDs, showed great potential for imaging and siRNA delivery *in vitro* with negligible cytotoxicity [82]. However, a more thorough investigation of their long-term cytotoxicity is necessary before they can be used *in vivo*.

 Gold nanoparticles (AuNPs) have emerged as a promising siRNA delivery carrier due to their excellent biocompatibility, ease of synthesis, high surface-to-volume ratio, and facile surface functionalization [83]. Recently, various types of AuNPs have been widely investigated for siRNA delivery. These include AuNPs functionalized with cationic quaternary ammonium or branched PEI, cationic lipid bilayer coated AuNPs, and oligonucleotide-modified AuNPs [83–85]. Gold nanorods also have the potential to deliver siRNA to target cells or tissues. The Prasad group developed gold nanorod-DARPP-32 siRNA complexes to target and reduce expression of the key proteins (DARPP-32, extracellular signal-regulated kinase (ERK), and protein phosphatase 1 (PP-1)) in the dopaminergic signaling pathway in the brain for therapy of drug addiction [86]. Using dark-field imaging and confocal microscopy, they demonstrated that the siRNA was efficiently delivered into dopaminergic neuronal (DAN) cells after treatment with the gold nanorod-siRNA conjugates. Moreover, the delivery of nanoplexes containing siRNA targeted to the DARPP-32 gene in DAN cells resulted in the silencing not only of DARPP-32, but also of other key downstream effector molecules in this pathway, such as ERK and PP-1, with greater efficiency than commercial transfection agents. Recently, Kim et al. reported that AuNPs stably functionalized with covalently attached oligonucleotides activate immune-related genes and pathways in human peripheral blood mononuclear cells, but not an immortalized, lineage-restricted cell line [87]. These later findings suggest that assessment of the toxic potential of engineered nanoparticles in immortalized, lineage-restricted cell lines may not predict their phenotypic effects in relevant biological systems.

## 5. Targeted Delivery

Significant advances have been made in the development of efficient siRNA delivery in nonviral vector systems, such as cationic lipids and polymers. However, a major problem with these approaches is that a large amount of siRNA has to be administered for efficient gene silencing. Moreover, cell type-specific targeting can prevent off-target effects, thus reducing the side effects of the therapeutics. A common approach for targeted delivery of siRNA to specific cells or tissues is conjugation to ligands such as antibodies, aptamers, and peptides which specifically bind to the corresponding moieties on target cells. Song and colleagues developed a protamine-antibody fusion protein for systemic and targeted siRNA delivery [88]. They fused protamine, a protein that binds nucleic acids, to a Fab directed against the human immunodeficiency virus type 1 (HIV-1) envelope protein and mixed the siRNA with the fusion protein. Treatment with the fusion protein mixed with siRNA targeted to the HIV-1 gag protein suppressed viral replication in infected primary T cells. Kumar et al. demonstrated T cell-specific siRNA delivery in a preclinical animal model [89]. In this study, a CD7-specific single-chain antibody was conjugated to oligo-9-arginine peptide (scFvCD7-9R) for T cell-specific siRNA delivery in humanized mice. Antiviral siRNAs complexed to scFvCD7-9R were shown to be delivered to naïve T cells and suppressed HIV replication in HIV-infected mice.

Nucleic-acid aptamers, which are normally selected from a large random-sequence pool to bind to a specific target molecule, have been explored for targeted siRNA delivery as an alternative to antibodies. Aptamers have advantages, such as high selective binding to proteins and receptors, ready-to-use chemical synthesis, process-compatible storability, and low immunogenicity [12]. McNamara II et al. have developed aptamer-siRNA chimeric RNAs for targeted delivery of siRNA [90]. The aptamer portions of the chimeras were introduced for specific binding to prostate-specific membrane antigen (PSMA), a cell-surface receptor overexpressed in prostate cancer cells and tumor endothelium, whereas the siRNA portion targeted the expression of survival genes. The chimeric RNA was demonstrated to bind only PSMA-expressing cells, resulting in depletion of siRNA target proteins and cell death. In addition, treatment with the chimeric RNA specifically inhibited tumor growth and mediated tumor regression in a xenograft model of prostate cancer. The aptamer-siRNA chimera is a promising targeted approach for siRNA delivery because RNA is not recognized by antibodies. However, more RNA and DNA aptamers must be developed for specific cancer or disease markers to expand the use of aptamer delivery approach. Recently, extensive studies have been performed to develop an RNA nanoparticle-based siRNA vector [91]. Packaging RNA is a 117-nt RNA molecule that constitutes one of the six packaging RNA subunits of the phi29 bacteriophage DNA packaging motor [92]. Chemically modified and folate receptor-targeted packaging RNA nanoparticles for siRNA delivery showed high *in vivo* stability with a blood half-life of 5 to 10 h and were retained in cancer tissue for more than 8 h. Tumor-targeted delivery and efficacy of gene silencing have also been in xenograft tumor models [92–94].

Another strategy for enhanced delivery of siRNA involves covalent conjugates to cell penetrating peptides (CPPs) or protein transduction domains [95]. The cationic nature of CPPs is crucial for their ability to bind and pass through the anionic cellular membrane. CPP conjugates of siRNA exhibited gene-silencing effects on target receptor proteins in various mammalian cell lines. However, conjugation of cationic peptides to anionic siRNA may neutralize and reduce the penetrating efficacy of these peptides [95]. In addition, CPP-siRNA conjugates may exhibit cytotoxicity caused by cell membrane perturbation or immunogenicity [96].

Recently, we have developed a new approach for targeted delivery and expression of siRNAs *in vivo* using DNA-based siRNA expression nanocassettes and receptor-targeted nanoparticles [97]. This new nanoparticle consists of an amphiphilic polymer-coated QD conjugated to 10 to 20 DNA nanocassettes that contain a U6 promoter and shRNA gene for *in vivo* siRNA gene expression following delivery to target cells. The nanoparticle was conjugated to the amino terminal fragment of urokinase plasminogen activator (uPA), which targets its cellular receptor, uPAR. This receptor is highly expressed in tumor, angiogenic endothelial, and stromal cells in many types of human cancers [98, 99]. Targeted delivery and gene-silencing efficiency of firefly luciferase siRNA nanogenerators were demonstrated in tumor cells and in animal tumor models. Moreover, delivery of survivin siRNA-expressing nanocassettes into tumor cells induced apoptotic cell death and sensitized cells to chemotherapeutic drugs. In cultured cells, the extent of targeted gene knockdown by survivin siRNA-expressing DNA nanocassettes using the uPAR-targeted nanoparticle delivery system was similar to that achieved with SV40-nuclear localization signal- (NLS-) mediated internalization of the QD-survivin siRNA nanocassettes. However, SV40-NLS-QD-siRNA nanocassettes could not be used for *in vivo* delivery due to their lack of specificity. These findings suggest that a receptor-targeted nanoparticle carrier allows efficient delivery into target tissues as well as intracellular delivery.

## 6. Clinical Trials

Currently, there are six cancer clinical trials underway using nanoparticle-based siRNA delivery, all in Phase I, evaluating the initial safety and utility of these treatments (Table 1). All the nanoparticle-formulated siRNA delivery systems for cancer therapy that are currently in clinical trials are based on polymers or liposomes. Since CALLA-01 was developed for the first siRNA phase I trial by Calando Pharmaceuticals, several other companies, including Tekmira, Alnylam, Silence Therapeutics, Marina, and others, have introduced siRNA nanoparticle products in either the preclinical or clinical phases. Silenseed Ltd (Jerusalem, Israel) initiated a phase I dose-escalation trial for siG12D LODER local drug eluter (LODER) (http://www.clinicaltrials.gov/ct2/show/NCT01188785). The siG12D LODER is a miniature biodegradable polymeric matrix that encompasses siRNA target to KRASG12D mRNA (siG12D) drug, designed to release the drug locally within a pancreatic tumor, for a prolonged period of 8 weeks. The siG12D LODER is injected into the patient's tumor with needle during an endoscopic ultrasound (EUS) biopsy procedure. The majority of pancreatic ductal adenocarcinomas involves mutations in the KRAS oncogene with the most common being G12D; therefore, administration of KRASG12D siRNA has the potential to silence KRAS, leading to apoptosis of the cancer cells and, thereby, slowing and halting tumor growth. In an upcoming Phase II study, a single dose of 3,000 *μ*g (eight 375-*μ*g siG12D LODERs) will be administered to patients with unresectable, locally advanced pancreatic cancer, in combination with chemotherapy treatment (http://www.clinicaltrials.gov/ct2/show/NCT01676259).

## 7. Conclusions and Future Perspectives

Since RNAi was discovered, various nonviral vector delivery systems for siRNA delivery have been explored extensively. Although significant advances have been made in the development of efficient *in vivo* siRNA delivery, there are still many challenges and barriers that must be overcome to achieve the ideal formulation in terms of selectivity, efficacy, and safety. Only a few nanoparticle-based siRNA delivery systems have been approved by the FDA and are in clinical trials for cancer therapy. Delivery systems can improve specificity of cancer cell targeting, prevent non-specific delivery of siRNA, and may also protect the siRNA during transport. Nanoparticles conjugated to the targeting ligand for effective siRNA delivery increase the chance of binding the tumor surface receptor; however, the process also increases the overall size of the nanoparticle. The PEG coating of nanoparticles reduces uptake by RES, resulting in enhanced circulatory half-life, but reduces targeting specificity because PEG molecules sterically disrupt selective conjugation. Thus, the selection of appropriate cell-specific targeting moieties and careful design of stable and potent nanoparticle delivery systems is required for future development.

Other major challenges for RNAi-based cancer therapeutics include controlling the specificity of the siRNA, minimizing off-target effects, increasing resistance to nuclease degradation, and avoiding immune responses such as *α*/*β* interferons, RNA-dependent kinase effects, and toll-like immunity. Chemical modification of the siRNA, such as inserting a 2′-*O*-methyl ribose in the nucleotide in the second position of the guide strand, could reduce silencing of most off-target transcripts with complementarity to the siRNA guide [100]. Dual-targeted siRNA drugs, such as ALN-VSP02, which targets VEGF and KSP, may reduce the potential for off-target gene silencing and increase the chances of knocking down the desired target.

Various nanoparticle-based delivery systems such as cationic lipids, polymers, dendrimers, and inorganic nanoparticles have been demonstrated to provide effective and efficient siRNA delivery *in vitro* and *in vivo*. Future studies must focus on the *in vivo* safety profiles of the various delivery systems, including undesirable immune stimulation and cytotoxicity. It is critical to develop safe, biocompatible, and biodegradable nanoparticle delivery systems for the clinical application of RNAi-based cancer therapeutics.

## Figures and Tables

**Table 1:** siRNA cancer therapeutics in clinical trials.

Drug	Company	Vehicle	Target	Disease	Delivery route	Phase	Stage
CALAA-01	Calando Pharma	Cyclodextrin nanoparticle, Transferrin, PEG	M2 subunit of ribonucleotide reductase	Solid tumors	IV	I	Ongoing, not recruiting

Atu027	Silence Therapeutics	Liposomes (Lipoplexes, Cationic lipid)	Protein kinase N3	Solid tumors	IV	I	Completed

ALN-VSP02	Alnylam Pharma	SNALP	VEGF and KSP	Solid tumors with liver involvement	IV	I	Completed
				Solid tumors	IV	I	Completed

TKM 080301	Tekmira Pharma	SNALP	Polo-kinase-1	Solid tumors	IV	I	Recruiting
				Solid tumors with liver involvement	IV	I	Completed

siRNA-EphA2-DOPC	M.D. Anderson Cancer Center	Liposomes (neutral liposomes)	EphA2	Solid tumors	IV	I	Not yet open

siG12D LODER	Silenseed Ltd	Polymer matrix (LODER polymer)	KRASG12D	Pancreatic ductal adenocarcinoma	EUS biopsy needle	I	Ongoing, recruiting
						II	Not yet open
